# Pharmaceutical regulatory harmonization in Africa: a scoping review of policy design, implementation approaches, and outcomes

**DOI:** 10.3389/fmed.2026.1812798

**Published:** 2026-04-23

**Authors:** Julius Niyibizi, John Mugisha, Brian Songa, Emmanuel Nizeyimana, Diane Badaga Isimbi

**Affiliations:** 1 Pharmacist, Kigali, Rwanda; 2 Southern Medical University, Guangzhou, China; 3 Rwanda Food and Drugs Authority, Kigali, Rwanda

**Keywords:** Africa, African Medicines Agency, implementation barriers, pharmaceutical regulatory harmonization, regulatory efficiency

## Abstract

**Background:**

Limited capacity in African national medicine regulatory authorities results in delayed registrations, regulatory inefficiencies, and the ongoing circulation of substandard and falsified medicines. The African Medicines Regulatory Harmonization initiative and regional mechanisms work to make medicine approval faster and fairer across Africa using convergent mechanisms. We aim to map and integrate evidence on pharmaceutical regulatory harmonization in Africa, concentrating on policy, implementation strategies, and reported outcomes, and identify barriers, facilitators, and evidence gaps to guide future research and policy formulation.

**Methods:**

This scoping review applied Arksey and O’Malley’s framework (Levac refinements) and PRISMA-ScR reporting standards. Systematic searches of PubMed, Scopus, Web of Science, Embase, and targeted gray literature identified English-language sources addressing policy design, implementation, governance, barriers/facilitators, and outcomes of pharmaceutical regulatory harmonization in Africa. Data from 19 included documents (2015–2025) were charted and synthesized via descriptive summaries and inductive thematic analysis.

**Results:**

The primary strategies included joint reviews, collaboration in Good Manufacturing Practice (GMP) inspections, regulatory reliance, standardized technical guidelines, and partnership-based capacity building. Documented improvements encompassed significant reductions in median joint review durations (e.g., EAC: 553–259 days), elevated application volumes, enhanced adherent GMP rates, improved Global Benchmarking Tool maturity levels, and economically viable regional recommendations. Major barriers were resource limitations, maturity differentials, delayed national uptake, weak binding reliance, coordination inefficiencies, and donor dependency; key facilitators included political will, partner support, International Medicines Regulatory Authority (IMRA) trust, and proven process efficiencies.

**Conclusion:**

Regional harmonization has produced significant regulatory efficiency and capability in East and Southern Africa. Regional harmonization has resulted in continued progress toward the Africa Medicine Agency necessities, enhanced political dedication, and diversified funding. Expedited adoption of standardized protocols, reinforced reliance mechanisms, and theory-based longitudinal assessments are needed to ensure equitable and sustainable access to quality pharmaceuticals.

## Introduction

Access to safe, effective, and quality-assured medical products is essential for public health, yet it relies on well-functioning regulatory systems. Numerous African national medicines regulatory authorities (NMRAs) encounter restricted capacity to execute essential responsibilities, leading to the proliferation of inferior and falsified products, which in turn causes detrimental public health and economic repercussions (1, 2). Ongoing constraints, including labor force scarcities, uneven technical competence, and budget limitations, hinder regulatory performance, resulting in inefficiencies in product evaluation, oversight, and prompt decision-making (1, 2).

Pharmaceutical regulatory harmonization has arisen as a strategic solution to these difficulties, seeking to enhance capacity, align standards internationally, minimize redundancy, and broaden access to quality-assured medication (3–5). The African Medicines Regulatory Harmonization (AMRH) initiative, initiated in 2009, stimulated regional frameworks such as the East African Community Medicines Regulatory Harmonization (EAC MRH), Southern African Development Community (SADC), and Economic Community of West African States Medicines Regulatory Harmonization (ECOWAS/WA-MRH). These enable joint work-sharing approaches, such as collaborative assessments and inspections (3, 6, 7). Stakeholders associate these joint procedures and convergence efforts with reduced regulatory workload, faster patient access to quality assured medicines, and improved efficiency. Continental efforts, including the establishment of the African Medicines Agency (AMA), build on regional experiences to further advance harmonization (1, 7, 8).

Of the eight officially recognized Regional Economic Communities (RECs) in Africa, six have established medicines regulatory harmonization initiatives under the AMRH framework: the East African Community (EAC-MRH), Southern African Development Community (SADC/ZaZiBoNa), Economic Community of West African States (ECOWAS/WA-MRH), Intergovernmental Authority on Development (IGAD, with emerging efforts), Economic Community of Central African States (ECCAS), and the recently launched North Africa Medicines Regulatory Harmonization (NA-MRH) initiative, which is the youngest and unites countries including Algeria, Egypt, Libya, Mauritania, Morocco, and Tunisia.

Despite these advancements, comparative and initiative-specific evaluations reveal substantial variation in implementation models, efficiencies, and performance across regions. Ongoing challenges include resource constraints, coordination difficulties, regulatory capacity differentials, and inconsistent national uptake of regional processes (1, 6, 7). The EAC MRH has shown improvements in registration timelines and inspection capacity; however, gaps still exist in achieving consistent and timely regulatory decision-making (2, 7). The evidence base remains fragmented and heterogeneous, with limited systematic synthesis of policy designs, practical implementation approaches, institutional and governance factors, barriers and facilitators, or measured outcomes.

This scoping review will map existing evidence on pharmaceutical regulatory harmonization in Africa, with a focus on policy design, implementation approaches, and reported outcomes. It will also identify implementation strategies; describe institutional, political, and contextual barriers and facilitators; examine outcomes related to regulatory efficiency, access to medicines, and system performance; assess the application of implementation science or policy frameworks; and highlight evidence gaps to guide future qualitative and policy analysis.

This scoping review maps existing evidence on pharmaceutical regulatory harmonization in Africa, focusing on (1) policy design and institutional arrangements; (2) implementation approaches and strategies; (3) barriers and facilitators; (4) reported outcomes related to regulatory efficiency, system performance, and access to medicines; and (5) evidence gaps to guide future research and policy.

## Methods

This scoping review was conducted in accordance with the methodological framework proposed by Arksey and O’Malley (9), with refinements recommended by Levac et al. (10) to enhance clarity and rigor. The review is reported following the Preferred Reporting Items for Systematic Reviews and Meta-Analyses extension for Scoping Reviews (PRISMA-ScR) guidelines (11). A protocol outlining the review methods was developed *a priori* and is available from the corresponding author upon request.

### Eligibility criteria

Sources of evidence were included if they met the following criteria: (1) addressed pharmaceutical regulation or regulatory harmonization; (2) focused on Africa or African regional bodies [e.g., African Union (AU), African Medicines Regulatory Harmonization (AMRH) program, African Medicines Agency (AMA), or Regional Economic Communities (RECs) such as the East African Community (EAC), Economic Community of West African States (ECOWAS), or Southern African Development Community (SADC)]; (3) discussed aspects of policy design, implementation approaches, governance, barriers/facilitators, or outcomes; (4) were published in English. No restrictions were placed on publication dates to capture the full historical and contemporary evidence base.

Sources were excluded if they (1) focused solely on clinical or pharmacological aspects without regulatory/policy content; (2) were unrelated to regulation (purely economic or epidemiological studies without regulatory focus) or (3) addressed non-African settings.

The Population-Concept-Context (PCC) framework guided eligibility: Population (national/regional regulatory authorities, stakeholders, or systems in Africa); Concept (pharmaceutical regulatory harmonization, including policy design, implementation, and outcomes); Context [African continental, regional (RECs), or national levels].

### Information sources

Academic databases searched included PubMed, Scopus, Web of Science, and Embase. Gray literature sources comprised websites and repositories of key organizations: the African Union (AU), the African Medicines Regulatory Harmonization (AMRH) program, the African Medicines Agency (AMA), the East African Community (EAC), ECOWAS, SADC, and selected National Medicines Regulatory Authorities. Additional sources were identified by hand-searching the reference lists of included studies and conducting forward citation searches of key publications, such as seminal AMRH or EAC reports.

### Search strategy

A comprehensive search strategy was developed iteratively by the review team to ensure sensitivity and breadth, drawing on the review questions and eligibility criteria (PCC framework). It combined controlled vocabulary (where applicable, e.g., MeSH terms in PubMed) and free-text keywords related to pharmaceutical/medicine/drug regulation, regulatory harmonization/convergence/integration/joint registration, medicine registration/market authorization, policy implementation, and Africa/African Union/Regional Economic Communities (e.g., EAC, ECOWAS, SADC). No filters were applied for publication date, study type, or language beyond the English inclusion criteria to maximize the retrieval of relevant academic and gray literature.

Example PubMed search string:

*[(“pharmaceutical regulation” OR “medicine regulation” OR “drug regulation” OR “medicines regulation”) AND (“harmonization” OR “harmonization” OR “regional integration” OR “joint registration” OR “mutual recognition”) AND (“Africa” OR “African Union” OR “East African Community” OR “ECOWAS” OR “SADC” OR “AMRH” OR “African Medicines Agency”)]*

Additional targeted strings included:

*“medicines regulation” AND implementation AND Africa*

*“African Medicines Regulatory Harmonization” OR “AMRH” OR “regulatory harmonization” AND Africa*

“East African Community” AND “regulatory harmonization”

Search strategies were adapted for each database (e.g., using appropriate syntax and field codes for Scopus, Web of Science, and Embase).

### Selection of sources of evidence

All records were imported into EndNote for deduplication. Title and abstract screening were performed independently by two reviewers (NJ and MJ), using predefined eligibility criteria, with conflicts resolved through discussion or consultation with a third reviewer (SB). The same two reviewers independently retrieved and assessed potentially relevant full texts. Disagreements were resolved through discussion, with a third reviewer arbitrating if needed. Reasons for exclusion at the full-text stage were documented. The study selection process is reported using a PRISMA-ScR flow diagram.

### Data charting process

The review team iteratively developed a standardized data charting form and pilot-tested it on 5–10 diverse sources to refine categories and ensure consistency. Charting was performed independently by two reviewers, with discrepancies resolved through discussion. The data were charted in Microsoft Excel. Key variables extracted included bibliographic details (author, year, title, source type); study/initiative characteristics [country/region, harmonization initiative (e.g., AMRH, EAC MRH)]; policy design elements; implementation strategies/modalities (e.g., joint assessment, work-sharing); institutional/governance arrangements; barriers and facilitators; reported outcomes (e.g., regulatory efficiency, access improvements, system performance); use of frameworks (implementation science or policy); and identified evidence gaps. Assumptions and simplifications (e.g., categorization of outcomes) were noted where applicable. No critical appraisal of individual sources was conducted, consistent with scoping review methodology.

### Synthesis of results

Charted data were synthesized using a combination of descriptive numerical summaries (e.g., frequency counts of initiatives by region, publication type, and year) and qualitative thematic analysis of implementation approaches, barriers/facilitators, and outcomes. Initiatives were mapped by region (continental, REC-specific, and national) and policy level. Thematic analysis followed an inductive approach, with themes developed iteratively from the data. Findings are presented narratively, with tables and figures (e.g., mapping of initiatives, PRISMA flow diagram) to enhance clarity. No meta-analysis or formal quality assessment was performed. The thematic analysis was conducted primarily by NJ, with iterative input from and validation by co-authors MJ, NE, BID, and SB.

## Results

### Selection of sources of evidence

**FIGURE 1:**
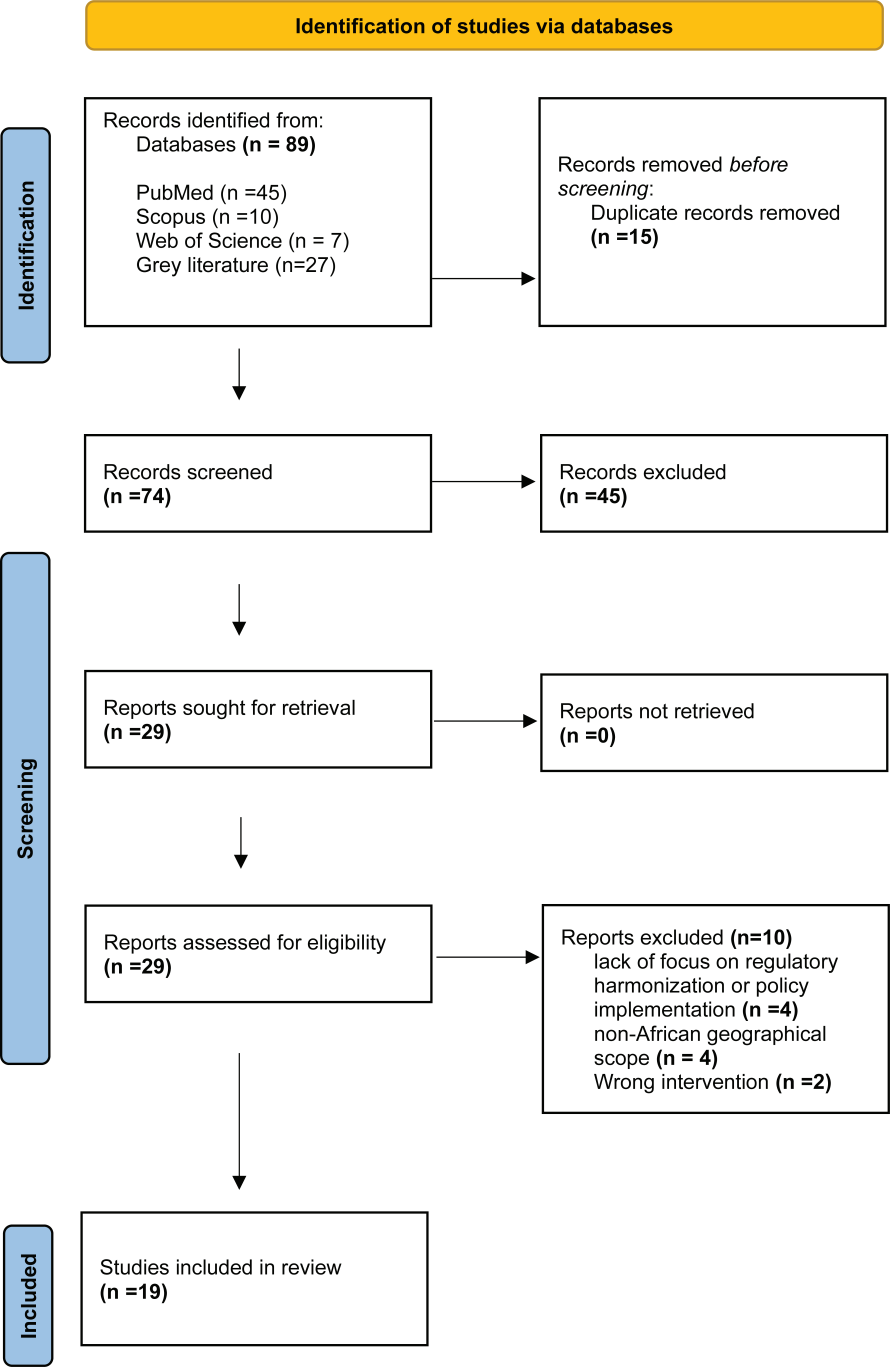
Characteristics of included studies using the PRISMA.

The search of academic databases and targeted gray literature sources identified 89 records. After removal of duplicates (*n* = 15), 74 unique records were screened by title and abstract. Of these, 45 were excluded, leaving 29 records for full-text assessment. After full-text review, 10 records were excluded for the following reasons: lack of focus on regulatory harmonization or policy implementation (*n* = 4), non-African geographical scope (*n* = 4), and wrong intervention or purely clinical/pharmacological content without a regulatory/policy component (*n* = 2). A total of 19 sources were included in the final synthesis. The study selection process is presented in Figure 1 (PRISMA-ScR flow diagram).

### Characteristics of included sources

The 19 sources included in this review were published between 2015 and 2025. The earliest document, although conducted in 2013, appeared in full form around 2015, while the most recent sources were published in 2025. Publication frequency exhibited a clear upward trend over this period: one source was published in each of 2015, 2016, 2018, and 2020; two sources appeared in 2021; two in 2023; six in 2024; and five in 2025 (Table 1).

**TABLE 1:** Characteristics of included sources (*n* = 19).

Article	Author/ year	Publication type	Study type (simplified)
1	Narsai et al. (12)	Peer-reviewed	Qualitative (literature + focus groups)
2	Ndomondo-Sigonda et al. (2)	Peer-reviewed	Mixed methods (interviews + quantitative indicators)
3	Narsai et al. (13)	Peer-reviewed	Comparative content analysis
4	Lahai et al. (14)	Peer-reviewed	Retrospective quantitative/qualitative review
5	Ncube et al. (15)	Peer-reviewed	Commentary/narrative review
6	Ncube et al. (16)	Peer-reviewed	Cross-sectional survey (census)
7	Suleman et al. (17)	Peer-reviewed	Mixed-methods comparative policy analysis
8	East African Community [EAC] Secretariat (18)	Gray literature (brochure)	Descriptive/promotional brochure
9	Lion’s Head Global Partners [LHGP] (19)	Gray literature (consultancy report)	Consultancy report/policy analysis
10	East African Community [EAC] Secretariat (20)	Gray literature (agreement)	Formal legal/policy document
11	Ncube et al. (21)	Peer-reviewed	Cross-sectional survey (CFIR)
12	Alfonso et al. (22)	Peer-reviewed	Cross-sectional NRA self-assessment (GBT)
13	Mashingia et al. (23)	Peer-reviewed	Retrospective review/timelines analysis
14	Sithole et al. (7)	Peer-reviewed	Mixed-methods comparative (questionnaires + interviews)
15	Kohler et al. (24)	Peer-reviewed	Policy analysis/desk-based assessment
16	Dube-Mwedzi and Suleman (25)	Peer-reviewed	Mixed-methods evaluation (surveys + desk review)
17	Ngum et al. (26)	Peer-reviewed	Mixed-methods evaluation (questionnaires)
18	Fimbo et al. (27)	Peer-reviewed	Historical policy analysis/desk review
19	Green et al. (28)	Peer-reviewed	Retrospective audit/quantitative comparison

Sixteen sources (84%) were peer-reviewed journal articles. Three sources (16%) were gray literature: one informational brochure from the EAC Secretariat (2020), one consultancy report commissioned by EAC and the World Bank (2016), and one formal inter-authority Cooperation Framework Agreement (2018).

Study designs were predominantly qualitative or mixed-methods. The most common approaches included qualitative policy analysis/narrative review (*n* = 5), cross-sectional surveys of NMRAs or stakeholders (*n* = 4), mixed-methods evaluations with questionnaires and interviews (*n* = 4), retrospective reviews or audits (*n* = 3), and comparative content/legislative analysis (*n* = 2). One source was a descriptive/promotional brochure without empirical data.

**FIGURE 2:**
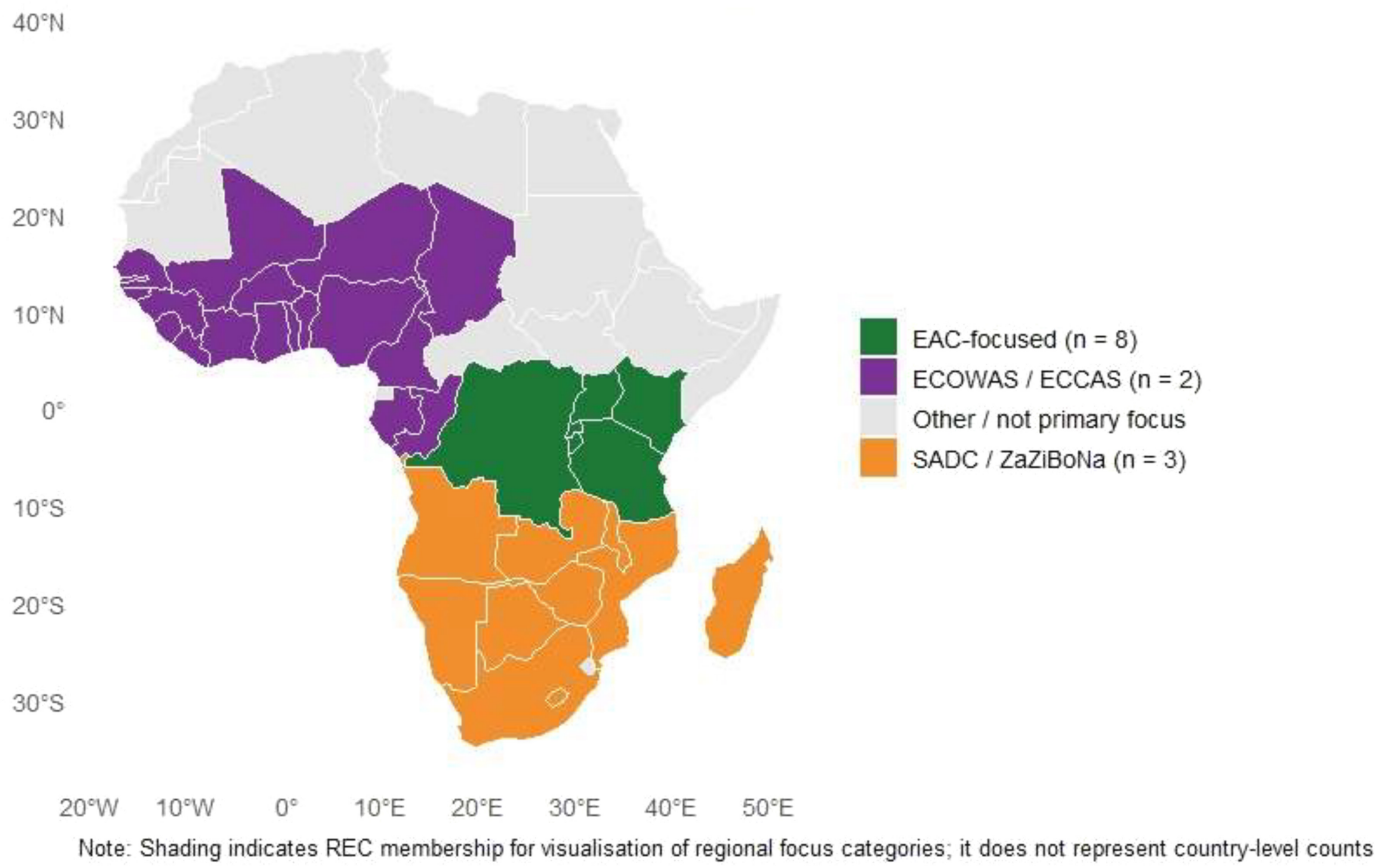
Primary regional focus of included sources (REC-level display). Some African countries are members of more than one REC (e.g., Tanzania and the Democratic Republic of Congo are part of both EAC and SADC; Kenya is part of EAC and IGAD). The shading reflects primary focus in included sources rather than exclusive membership. Shading indicates REC membership for visualization of regional focus categories; it does not represent country-level counts.

Geographic and regional focus varied: eight sources (42%) concentrated on the East African Community (EAC) Partner States (six or seven countries depending on document date); four (21%) addressed continent-wide issues across African Union (AU) Member States (including surveys of 45 or 55 countries); three (16%) focused on Southern African Development Community (SADC)/ZaZiBoNa (including one with 7 NRAs and one with 16 countries); two (11%) covered West and Central Africa (ECOWAS/ECCAS, including one with 10 NRAs); one (5%) was national (Sierra Leone); one (5%) national with comparative elements (Ethiopia + selected African countries and EU benchmark); and two (11%) included multi-REC comparisons (EAC, SADC/ZaZiBoNa, ECOWAS) (Figure 2). One source included a comparative element with Europe (EMA).

### Pharmaceutical regulatory harmonization initiatives in Africa

**FIGURE 3:**
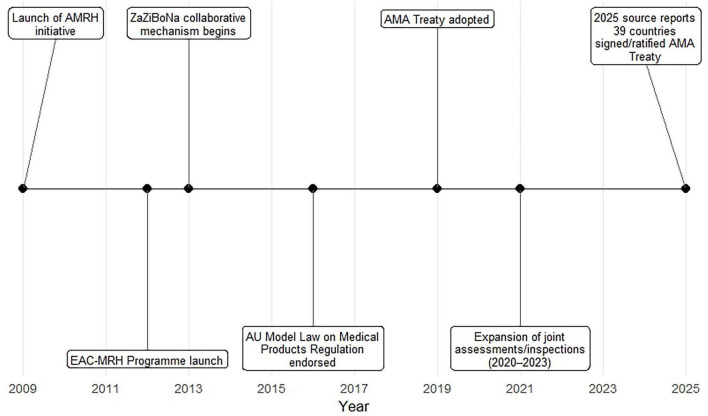
Timeline of key milestones in African pharmaceutical regulatory harmonization (2009-2025). AMRH, African Medicines Regulatory Harmonization; EAC-MRH, East African Community Medicines Regulatory Harmonization; AMA, African Medicines Agency.

The African Medicines Regulatory Harmonization (AMRH) initiative (established in 2009) was the most frequently referenced overarching framework, explicitly mentioned in 14 sources (74%). Regional work-sharing initiatives were prominent: the East African Community Medicines Regulatory Harmonization (EAC-MRH) program or joint procedures appeared in 11 sources (58%); the Southern African Development Community/ZaZiBoNa collaborative mechanisms in 4 sources (21%); and the ECOWAS/WA-MRH or ECCAS regional approaches in 2 sources (11%). The AMA, including its treaty (adopted 2019), was discussed in 6 sources (32%). The AU Model Law on Medical Products Regulation (endorsed in 2016) was addressed in 4 sources (21%) (Figure 3). Other mentions included joint Good Manufacturing Practice (GMP) inspections (*n* = 3; 16%), and national regulatory evolution (e.g., Tanzania TMDA/TFDA; Ethiopia FMHACA).

Core mechanisms across major initiatives included joint dossier assessments and work-sharing for marketing authorization (most common in EAC-MRH and ZaZiBoNa), joint GMP inspections (physical and desk-based, primarily EAC), regulatory reliance on regional recommendations, adoption of harmonized technical guidelines [e.g., Common Technical Document (CTD), labeling, GMP standards], capacity building via twinning models, and domestication of the AU Model Law to enable autonomous NMRAs and regional convergence.

### Implementation approaches and strategies

The most frequently reported implementation strategies were joint assessment/work-sharing of marketing authorization applications (*n* = 10 sources; 53%), joint GMP inspections or desk reviews (*n* = 5; 26%), regulatory reliance on regional/partner assessments (*n* = 9; 47%), adoption and use of harmonized guidelines (registration, GMP, labeling, etc.) (*n* = 8; 42%), and capacity building through twinning, training, or peer support (*n* = 5; 26%). Other strategies included domestication of the AU Model Law into national legislation (*n* = 4; 21%), implementation of Quality Management Systems (QMS) with ISO 9001:2015 certification (*n* = 2; 11%), and use of the WHO GBT for maturity assessment and Institutional Development Plans (*n* = 3; 16%). Voluntary participation via expressions of interest or consent for joint processes was noted in EAC-MRH contexts.

Named implementation science or policy frameworks were rarely applied. Only one source explicitly used the Consolidated Framework for Implementation Research (CFIR) to analyze the AU Model Law domestication. One source adapted the WHO 2018 Pharmaceutical System Transparency and Accountability Assessment Tool for the regulatory-procurement interface. All other sources (*n* = 17; 89%) reported no named theoretical or implementation science framework.

### Barriers and facilitators to implementation

Barriers and facilitators were described across most sources. The most frequent barriers were resource constraints (workforce, funding, technical capacity) and differential NRA maturity levels (*n* = 12 sources each; 63%), delays in national registration/uptake after joint recommendations (*n* = 8; 42%), lack of full reliance/mutual recognition or binding regional decisions (*n* = 7; 37%), coordination and process inefficiencies (*n* = 6; 32%), slow legislative reform or domestication (*n* = 6; 32%), and donor dependence/lack of financial sustainability (*n* = 5; 26%). Other barriers included varying timelines/fees, limited transparency in the procurement-regulation interface, and over-registration of non-essential medicines.

The most frequent facilitators were strong political commitment/will (including AU mandates and treaty signing) (*n* = 10; 53%), donor/partner support (funding, technical assistance from WHO, World Bank, Gates Foundation, EMA) (*n* = 9; 47%), trust-building and collaboration among NMRAs (*n* = 8; 42%), demonstrated benefits of joint processes (efficiency, reduced duplication) (*n* = 7; 37%), harmonized guidelines and standards (*n* = 6; 32%), and capacity building via twinning/joint activities (*n* = 5; 26%) (Table 2).

**TABLE 2:** Most frequently reported barriers and facilitators to implementation.

Rank	Barriers	Number of sources	Facilitators	Number of sources
1	Resource constraints (workforce, funding, capacity)	12	Strong political commitment/will	10
2	Differential NRA maturity levels	12	Donor/partner support (funding, technical assistance)	9
3	Delays in national registration/uptake	8	Trust-building and collaboration among NMRAs	8
4	Lack of full reliance/mutual recognition	7	Demonstrated benefits of joint processes	7
5	Coordination and process inefficiencies	6	Harmonized guidelines and standards	6
6	Slow legislative reform/domestication	6	Capacity building via twinning/joint activities	5
7	Donor dependence/lack of financial sustainability	5	–	–

### Reported outcomes

Reported outcomes were grouped into regulatory efficiency, system performance/maturity, access/quality of medicines, and other domains.

Regulatory efficiency: multiple EAC-MRH sources reported reductions in median joint review times (e.g., from 553 days in 2015 to 259 days in 2023) and national registration delays (median 7 months post-recommendation vs. target 3 months) (26). Applications increased (9 in 2015 to 44 in 2023) (26). ZaZiBoNa reported a low cost per joint recommendation (USD 2,768) and a 64.7% registration rate after joint assessment (152/235 products) (25). ISO 9001:2015 implementation in one NMRA marked an increase in the detection of substandard and falsified (SF) medicines (14).

System performance/maturity: WHO GBT assessments showed improvements (e.g., one NMRA from ML-1 in 2016 to higher levels by 2021, especially in QMS and pharmacovigilance) (14, 22). EAC joint GMP inspections achieved 65% compliance (24/37 facilities received 3-years certificates) (23). Four EAC NMRAs attained ISO 9001:2015 certification (23). Several sources noted that strengthened registration and inspection systems were supported by harmonized guidelines (2, 27).

Access/quality of medicines; high proportions of essential medicines remained unregistered in EAC countries (Kenya 28%, Tanzania 50%, Uganda 40%), while non-essential products dominated registrations (58%–71%) (28). Joint processes were linked to improved access to quality-assured medicines and reduced SF products. Procurement-regulation interface assessments highlighted weak transparency and enforcement as barriers to quality-assured supply (24). Other Positive perceptions of joint processes by NMRAs and moderate industry satisfaction were common (7, 25). Continental progress included AMA Treaty signatures/ratifications by 39 countries (as of May 2025, in one source) (26).

### Evidence gaps

The included sources revealed several evidence gaps. Named implementation science or policy frameworks were applied in only two sources. Most documents lacked direct cross-REC comparative analyses beyond broad AMRH overviews. Long-term outcomes (e.g., post-AMA operationalization impacts, sustained financial sustainability, or health outcome effects) remains largely absent from literature Given that the AMA is still in early operational stages (e.g., recent pilot continental listing processes under the AMRH program, headquarters in Kigali, and ongoing staffing), it is premature to expect extensive post-operationalization data. Francophone and non-EAC/non-SADC regions remained underrepresented. Quantitative data on access improvements (beyond registration timelines) and rigorous evaluations of reliance models were limited. These gaps highlight opportunities for future qualitative, longitudinal, and theory-informed research on regulatory harmonization in Africa.

## Discussion

This scoping review maps the developing framework of pharmaceutical regulatory harmonization in Africa, emphasizing significant progress propelled by the African Medicines Regulatory Harmonization (AMRH) initiative since 2009, along with following regional endeavors like the EAC-MRH, ZaZiBoNa (SADC), and evolving ECOWAS/WA-MRH mechanisms. These programs have facilitated collaborative work-sharing, joint evaluations, reliance models, standardized guidelines, and capacity-building efforts, resulting in quantifiable enhancements in regulatory efficiency and system maturity, especially in more developed areas such as East and Southern Africa.

Significant results demonstrate that collaborative procedures have shortened assessment timelines; For example, median joint review durations in the EAC have markedly reduced over periods with associated studies revealing that approvals for specific products (generics and WHO prequalified) averaged below 65 days in Uganda from 2020 to 2023, alongside overall enhancements in national registration delays (2, 23, 26, 29). However, post-recommendation national registration delays persist (median 7 months versus the target of 3 months (26), likely due to national-level administrative bottlenecks, resource constraints, and incomplete reliance mechanisms rather than the harmonization initiative itself causing these delays. This underscores the need for stronger national adoption of regional recommendations to fully realize efficiency benefits for patients. Application volumes have increased markedly, resulting in improved GMP inspection capability (e.g., 65% compliance in EAC joint inspections) (23), enhanced identification of inferior and counterfeit pharmaceuticals through quality management systems (14), and economical regional recommendations in ZaZiBoNa at USD 2,768 with a 64.7% registration rate post-assessment [152/235 products] (25). The low cost per joint recommendation in ZaZiBoNa demonstrates the economic viability of work-sharing models in reducing duplication and resource demands across NMRAs, while the moderate registration rate indicates incomplete national uptake; crucially, applicants must still pay separate national marketing authorization fees, meaning the joint process delivers regional-level savings but requires streamlined national follow-up to translate into equitable access improvements. These achievements correspond with overarching objectives of enhancing access to quality-assured pharmaceuticals and facilitating continental integration through the AMA.

System performance and maturity have also advanced in targeted areas. WHO GBT assessments have shown improvements (e.g., one NMRA progressing from ML-1 in 2016 to higher levels by 2021, particularly in QMS and pharmacovigilance) (14, 22), and four EAC NMRAs have attained ISO 9001:2015 certification (23). These gains align temporally with capacity-building under regional initiatives (e.g., joint training, reliance, and harmonized standards), though direct causation remains challenging to establish amid concurrent national reforms and global WHO support. Similarly, ISO 9001:2015 implementation in one NMRA was associated with increased detection of substandard and falsified medicines (14); while suggestive of quality surveillance benefits, this is limited to a single authority, lacks statistical robustness for generalization, and cannot be solely attributed to regional harmonization, as national efforts likely contribute. Several sources noted that strengthened registration and inspection systems were supported by harmonized guidelines (2, 27). Overall, these developments indicate that harmonization contributes to incremental system strengthening, particularly where political commitment and partner support enable sustained implementation, though uneven progress across regions highlights the importance of addressing maturity differentials for continent-wide impact.

Notwithstanding these gains, enduring obstacles highlight the inconsistent execution throughout the region. Resource limitations, varying regulatory maturity levels, delays in national adoption of regional proposals, restricted binding reliance or mutual recognition, coordination inefficiencies, slow legislative domestication, and dependency on donors persist as significant problems (7, 16, 22). These challenges are exacerbated by the inadequate representation of specific geographical areas (e.g., Francophone and Central Africa) and the scarcity of long-lasting outcome data beyond deadlines and registration rates. Francophone and non-EAC/non-SADC regions remained underrepresented, likely due to the English-language inclusion criterion and publication bias favoring English sources, and although the search was comprehensive, relevant francophone studies (e.g., from ECOWAS or ECCAS) in French journals or gray literature may have been missed; future reviews should consider multilingual searches to address this limitation. Broader literature on regulatory convergence (beyond the included sources) suggests that sustained political will, diversified funding, and clear governance mechanisms are essential for overcoming such structural obstacles, as observed in more mature models like the European Medicines Agency (EMA). In the African context, these factors will be critical for transitioning from regional pilots to equitable, sustainable continental effects under the AMA.

The limited utilization of implementation science frameworks (with just two sources specifically employing named models such as CFIR) (21) signifies a significant deficiency, as does the lack of robust cross-REC analyses, longitudinal assessments of health outcomes, and evidence regarding the sustainability of finances following donor support. The latest developments demonstrate the importance of this review: as of December 2025, the AMA Treaty has been approved by 56.4% of African Union Member States (with a head office opened and functioning in Kigali, Rwanda, and continuous efforts for comprehensive full continental coverage) (30). Transitioning from AMRH as a program to AMA as its institutional successor, accompanied by staged implementation plans, indicates a move toward enhanced centralized coordination; however, issues related to sovereignty, legal alignment, and resource equity remain.

In summary, while regional harmonization has produced measurable efficiency improvements and capacity advancements in certain areas, attaining equitable, sustainable, and continent-wide effects necessitates the resolution of structural obstacles through enhanced political commitment, diverse funding, expedited adoption of harmonized standards (which includes the AU Model Law), and increased dependence on regional and continent-wide results. The evidence base, while expanding (with publication patterns sharply increasing in 2024–2025), remains fragmented and primarily descriptive, constraining deeper causal understanding. Rigorous, longitudinal, and theory-informed evaluations will be essential to guide the AMA’s evolution and ensure timely access to quality-assured medicines for all Africans.

## Conclusion

This scoping review underscores significant progress in the harmonization of pharmaceutical regulations in Africa through AMRH and regional efforts such as EAC-MRH and ZaZiBoNa, resulting in shorter review timeframes, increased capacity, and higher regulatory efficiency. Ongoing obstacles, such as resource limitations, varying levels of maturity, postponed national adoption, and restricted reliance, persist in hindering equal and sustained impact across the continent. With the African Medicines Agency advancing, stronger political commitment, diversified funding, accelerated domestication of harmonized standards, and rigorous, theory-informed evaluations are critical to realizing timely access to quality-assured medicines for all Africans.

## Data Availability

The original contributions presented in this study are included in the article/supplementary material, further inquiries can be directed to the corresponding authors.
